# Acute Lethal Toxicity of Heavy Metals to the Seeds of Plants of High Importance to Humans

**DOI:** 10.1007/s00128-018-2382-9

**Published:** 2018-06-19

**Authors:** Kamila Pokorska-Niewiada, Monika Rajkowska-Myśliwiec, Mikołaj Protasowicki

**Affiliations:** West Pomeranian University of Technology in Szczecin, Papieża Pawła VI 3, 71-459 Szczecin, Poland

**Keywords:** LC50, Phytotoxicity, Toxicity test, Heavy metals, Seed germination

## Abstract

Laboratory experiments were conducted to assess the effects of highly toxic and dangerous metals (Pb, Cd, Hg) and essential microelements (Cr, Cu, Ni, Zn) on the seed germination process in vetch and eight plant species of major importance to human consumption. The degree of metal toxicity was determined on the basis of acute LC50 values calculated by means of two different methods. All the metals were found to inhibit the germination process, but in a varying, species-dependent degree. Of all the plant seeds under study, the most sensitive to the presence of the examined metals during germination were the seeds of cress (Cu, Pb, Hg), rye (Ni, Zn, Cd) and barley (Cr), while vetch (Cr, Ni, Zn, Cd, Pb, Hg), rape (Cr, Cu) and pea (Ni, Cd) were found to be least affected.

Due to progressive urbanization and modern agricultural practices, environmental pollution with heavy metals is on the increase. Metals come from the natural weathering of the earth’s crust, industrial discharge, pest or disease control agents applied to plants, urban runoff, mining, soil erosion, sewage effluents, air pollution fallout and other sources. Plants can be affected directly by air pollutants, as well as indirectly through the contamination of soil and water (Fargašová 1994). Heavy metals and other elements are absorbed by plants in ionic form. They are toxic to the life processes of plants, mainly through their interaction with the functional groups of molecules in the cell, especially proteins and polynucleotides. The presence of heavy metals like nickel, cobalt, cadmium, copper, lead, chromium and mercury in air, soil and water can cause bioaccumulation which affects the entire ecosystem and has harmful health consequences in all life forms. In higher concentrations, metals and chemicals hamper plant germination, growth and production, which are mainly associated with the physiological, biochemical and genetic elements of the plant system (Sethy and Ghosh 2013). As members of food chains, plants may pose risk to humans and animals alike through contamination of food supplies (Fargašová 1994; Zhou et al. 2014).

In recent years, there has been a significant increase in the study of the effects of soil pollution on plants, including observation of seed germination and initial stages of development (Smreczak and Maliszewska-Kordybach 2003; Li et al. 2005). Plants show a wide variation in sensitivity to metal contamination. Some of them are hyperaccumulators while others display great sensitivity to pollutants. Hyperaccumulators are an interesting group of plants which manage to survive under extreme environmental conditions (Tangahu et al. 2011). From among the tested plant species, winter rape (*Brassica napus* L.) belongs to this group. Sensitive species, including indicator species like barley (*Hordeum vulgare* L.) used in biotests for high concentrations of elements, are prone to structural damage and disturbances in biochemical and physiological processes, leading to growth retardation and even cell death. These biotests have many advantages: they are cheap and easy to use, do not require expensive laboratory equipment, are easy to observe, and produce repeatable results (Smreczak and Maliszewska-Kordybach 2003).

The aim of this study was to compare how different concentrations of heavy metals, both essential (Cu^2+^ Zn^2+^, Cr^3+^, Ni^2+^) and non-essential (Pb^2+^, Cd^2+^ and Hg^2+^), affect seed germination ability in plant species of great economic importance. In Poland, in 2016, cereals accounted for 70% of the total sown area, oilseeds (including oilseed rape) – 10.2%, pulses (including vetch and pea) – 3%, and vegetables (including carrots) – 2%. The cereal species analyzed in this study, i.e. wheat, barley, rye and oats, represented 31.9%, 12.4%, 10.5% and 6.4% of the total area sown with cereals, respectively. Regarding oilseeds, up to 95.5% of their total acreage was sown with rape and turnip rape. In terms of world production of selected agricultural products in 2014, was Poland ranked as follows: wheat – 15th; rye – 3rd; barley – 12th; oat – 3rd; rape – 7th (Statistical Yearbook of Agriculture 2016). The toxicity of the analyzed metal cations was compared based on the calculated LC50 values. Ultimately, this study was also carried out to select the most suitable plant species which could probably germinate efficiently in heavily contaminated areas.

## Materials and Methods

The material used for the study included seeds of vetch (*Vicia sativa* L.) and other plants of direct importance for human consumption, such as rye (*Secale cereale* L.), wheat (*Triticum aestivum* L.), barley (*Hordeum vulgare* L.), oat (*Avena sativa* L.), winter rape (*Brassica napus* L.), pea (*Pisum sativum* L.), carrot (*Pisum sativum* L.), and cress (*Cardamine pratensis* L.). The concentrations of individual metals (provided as nitrate salts) were determined by diluting the standard solutions (Merck KGaA, Darmstadt, Germany). The concentration was dependent on the phytotoxicity of the particular metal, its permitted content in light soils (Regulation of the Minister of Environment, Journal of Laws of 2016, item 1395), and the results of initial germination tests for selected concentrations. Eight metal concentrations were used in each germination test and the tests were carried out using triplicate samples. The seeds in the amount of 100 were placed on double-layered filter papers (3 mm, Whatman, Maidstone, UK), wetted with 10 mL of double-distilled water (ddH_2_O) or 10 mL of metal solutions in 12-cm Petri dishes. The seeds were not pre-soaked or sterilized. Petri dishes were covered and incubated at 22 ± 2°C.

The seeds were scored as germinated when the breakage of the seed coat was visible. Germination inhibition (%) was determined by reference to the control samples and the LC50 values were calculated using Trevan’s and Kärber’s methods described by Saganuwan (2016).

Statistical analyses, including variance analyses, were carried out using Statistica 13.1 software (StatSoft Inc.). Significant differences between the calculated mean values of LC50 for each species were determined using Duncan’s multiple range test at the significance level of *p* ≤ 0.05.

The nominal values of metal concentrations were verified using a sequential ICP-AES spectrometer (Yobin Yvon JY-24) equipped with a Meinhard TR 50-C1 nebulizer. The following spectrometer parameters were used: frequency, 40.68 MHz; power, 1.0 kW; demountable quartz torch, plasma flow, 12.0 L/min; nebulizer gas, Ar, 1.0 L/min; nebulizer pressure, 3.5 bar; sample flow rate, 1.1 mL/min; holographic grating, 2400 g/mm; linear dispersion better than 0.3 nm/mm. The criteria used to assess the analytical procedures were as follows: detection and quantification limits (LOD and LOQ respectively), linearity, sensitivity, recovery, and precision. LOD and LOQ were determined using the standard deviation of the blank signal multiplied by three and six, respectively. The limits of detection for individual elements are shown in Table 1. For each metal, a 7-point calibration curve was prepared using appropriate dilutions of standard solutions (in the range of 0–250 mg/L). Three measurements were taken for each of the 7 points. For the first analysis the curves were generated five times and, on this basis, the average slopes of the calibration curves and calibration coefficients were determined for each metal. The values of the calibration coefficients were ≥ 0.995. Calibrations were repeated every 10 samples (test solutions) to check the quality of determinations. Test solutions with concentrations higher than 250 mg/L were diluted. When compared with the nominal concentrations, the measured metal concentrations in the prepared solutions showed good accuracy, with recovery rates ranging between 92%–107%. A statistical analysis confirmed no significant differences between the measured and nominal concentrations (Duncan’s test, *p* ≤ 0.05).

**Table 1:** Limits of detection for aqueous solutions of single elements

Metal	Wavelength (nm)	LOD (µg/L)
Cr	283.6	3.2
Cu	327.4	2.6
Ni	231.6	5.1
Zn	213.9	1.1
Cd	228.6	0.9
Pb	283.3	15.1
Hg	523.7	9.5

## Results and Discussion

Heavy metals like lead, nickel, cadmium, copper, cobalt, chromium, and mercury are major environmental pollutants that have toxic effects on plants. Heavy metal stress affects plant physiology, which poses serious threats to the agro-ecosystems and results in reduced productivity (Sethy and Ghosh 2013).

In the available literature, authors usually refer to 1 to 5 metals and rarely to more than one plant species (Fargašová 1994; Peralta et al. 2001; El-Ghamery et al. 2003; Li et. al. 2005). This research is concerned with the effect of seven different metals on the germination of eight crop plants vital for humans, and vetch – a leguminous fodder plant, important as a protein source for animals and as a plant that improves soil fertility. The relations between seed germination inhibition (%) in the selected plant species and different concentrations of essential and toxic heavy metals, are presented in Tables 2 and 3. The level of seed germination in the control samples for each species was very high (> 97%), which proves the high quality of the tested material. Seed germination power was found to decrease with the increasing concentrations of ions of each metal, compared with a substrate saturated with distilled water (control). Similar observations for Ni and Cu ions were made in alfalfa (*Medicago sativa* L.), which is grown for green fodder, hay or silage (Peralta et al. 2001). Toxic effects on the germination process were also reported by other authors, e.g. El-Ghamery et al. (2003) – the effect of zinc on wheat seeds, or Tao et al. (2015) – different levels of tolerance of six pulse crops to cadmium. Taking into consideration the lowest metal concentration that caused significant inhibition of germination (min. 20%), plant species with the highest sensitivity to each metal were selected. According to Kranner and Colville (2011), seed germination depends on the taxon, population, and dose of metal, which is also confirmed by this research. The results indicate that significant germination inhibition (min. 20%) at different metal concentrations depends on the species. The lowest concentrations of metals at which a significant reduction in seed germination (min. 20%) was observed were as follows: Cr – barley, oat (at 10 mg/L); Cu – wheat (5 mg/L), oat (between 10 and 20 mg/L), rye and pea (20 mg/L); Ni – rye (30 mg/L); Zn – rye (1 mg/L), wheat (10 mg/L); Cd – rye (1 mg/L) and carrot (5–10 mg/L); Pb – oat (1 mg/L), rye and wheat (10–20 mg/L); Hg – wheat (1 mg/L), rye (1–10 mg/L).

**Table 2:** Inhibition of germination (%) in the test plants under varied concentrations of Cr, Cu, Ni, and Zn

Wheat	Barley	Rye	Oat	Rape	Carrot	Pea	Cress	Vetch
mg/L (% of germination inhibition)								
Cr								
0 (0.7)	0 (0)	0 (0)	0 (0)	0 (0)	0 (0)	–	0 (0)	0 (2.0)
50 (36.9)	10 (22.4)	50 (34.3)	5 (15.0)	100 (0.7)	50 (7.7)		50 (1.7)	50 (5.4)
100 (46.0)	20 (36.6)	100 (58.7)	10 (21.0)	200 (2.7)	100 (21.3)		100 (3.0)	150 (14.6)
150 (63.1)	50 (46.8)	150 (87.3)	20 (24.0)	400 (6.7)	150 (35.3)		150 (4.0)	250 (17.3)
200 (83.6)	100 (90.8)	200 (90.7)	50 (27.7)	600 (7.3)	200 (61.7)		200 (5.3)	300 (18.7)
250 (93.3)	150 (95.3)	250 (92.7)	100 (48.7)	800 (7.3)	250 (67.5)		250 (14.7)	600 (21.8)
300 (94.0)	200 (96.3)	300 (96)	150 (68.3)	1100 (11.0)	300 (95.7)		300 (26.3)	900 (23.4)
400 (97.0)	250 (98.6)	400 (96.0)	200 (91.7)	1300 (51.0)	400 (98.5)		400 (60.0)	1100 (54.4)
500 (99.3)	300 (100)	500 (100)	250 (99.7)	1600 (98.7)	600 (100)		500 (96.7)	1500 (97.3)
Cu								
0 (0)	0 (0)	0 (0)	0 (1.0)	0 (0)	0 (0)	0 (2.0)	0 (0)	0 (1.3)
5 (22.0)	5 (1.7)	5 (2.3)	5 (7.7)	20 (2.0)	20 (5.3)	20 (22.1)	5 (1.7)	5 (1.7)
10 (29.0)	10 (3.3)	10 (5.0)	10 (18.5)	80 (2.3)	40 (26.3)	80 (38.1)	10 (2.7)	10 (2.4)
20 (32.7)	20 (4.7)	20 (21.3)	20 (25.6)	160 (5.7)	50 (47.0)	160 (57.1)	20 (4.0)	20 (5.1)
40 (43.3)	50 (12.7)	40 (28.0)	40 (37.0)	240 (13.0)	60 (67.3)	240 (82.7)	30 (13.3)	40 (7.4)
80 (49.3)	80 (16.3)	50 (30.3)	80 (41.8)	480 (37.3)	80 (80.7)	480 (90.1)	35 (22.0)	80 (14.2)
160 (55.0)	160 (59.3)	100 (78.3)	160 (81.8)	700 (86.0)	100 (89.3)	700 (96.3)	40 (53.7)	160 (43.6)
240 (98.3)	240 (93.7)	200 (98.7)	240 (96.6)	850 (94.7)	150 (97.0)	850 (99.3)	50 (93.3)	240 (88.9)
480 (100)	480 (100)	400 (100)	480 (99.7)	1000 (98.7)	200 (100)	1000 (100)	60 (100)	480 (99.0)
Ni								
0 (2.3)	0 (0.7)	0 (0)	0 (1.0)	0 (0)	0 (0)	0 (0.0)	–	0 (0)
30 (10.2)	30 (5.4)	30 (27.7)	30 (10.8)	30 (4.7)	20 (10.3)	50 (1.0)		30 (4.0)
50 (24.2)	50 (17.8)	50 (45.3)	50 (16.8)	50 (14.0)	50 (18.0)	100 (1.7)		100 (7.0)
100 (27.6)	100 (26.5)	100 (70.3)	100 (27.3)	100 (38.7)	100 (36.0)	200 (14.0)		200 (14.7)
150 (41.0)	150 (52.3)	150 (83.0)	150 (55.2)	200 (89.3)	150 (41.3)	250 (39.7)		350 (50.7)
200 (53.9)	200 (78.5)	200 (88.0)	200 (81.8)	300 (91.3)	200 (56.7)	350 (46.3)		450 (62.7)
250 (64.2)	250 (87.2)	250 (94.0)	250 (91.6)	450 (98.3)	250 (75.7)	450 (54.7)		550 (76.7)
350 (87.0)	350 (99.3)	350 (98.7)	350 (99.0)	550 (99.0)	300 (91.3)	550 (70.0)		700 (83.3)
450 (100)	450 (100)	450 (100)	450 (100)	700 (100)	400 (100)	750 (97.7)		800 (99.7)
Zn								
0 (2.3)	0 (0)	0 (2.3)	0 (0)	0 (0)	0 (0)	0 (0)	0 (0)	0 (0)
10 (20.8)	10 (13.0)	1 (46.1)	10 (27.0)	10 (0.3)	50 (3.0)	50 (17.3)	5 (4.3)	10 (2.3)
20 (26.6)	20 (25.3)	5 (54.6)	20 (30.0)	100 (1.0)	100 (14.3)	100 (16.7)	10 (16.7)	100 (2.7)
50 (44.4)	50 (47.7)	10 (64.2)	50 (38.3)	150 (2.0)	150 (32.7)	200 (20.7)	20 (20.7)	150 (3.7)
100 (53.6)	100 (52.3)	20 (85.3)	100 (45.7)	250 (15.3)	200 (44.7)	250 (27.0)	50 (79.3)	300 (3.7)
150 (90.8)	150 (73.0)	50 (90.4)	150 (60.0)	300 (36.0)	250 (54.7)	300 (39.7)	100 (91.7)	500 (5.7)
200 (97.6)	200 (92.3)	100 (93.5)	200 (88.3)	500 (77.7)	300 (78.3)	500 (53.3)	150 (95.7)	750 (63.0)
250 (99.0)	250 (99.0)	150 (97.3)	250 (99.7)	750 (95.0)	400 (96.7)	750 (80.1)	200 (99.0)	900 (78.0)
300 (100)	300 (100)	300 (100)	300 (100)	900 (99.0)	600 (100)	900 (99.7)	250 (100)	1100 (99.7)

n = 3, – not studied

**Table 3:** Inhibition of germination (%) in the test plants under varied concentrations of Cd, Pb, and Hg

Wheat	Barley	Rye	Oat	Rape	Carrot	Pea	Cress	Vetch
mg/L (% of germination inhibition)								
Cd								
0 (0)	0 (0)	0 (0)	0 (0)	0 (0)	0 (0)	0 (0)	0 (0)	0 (0)
1 (5.6)	10 (2.3)	1 (26.3)	10 (1.7)	1 (3.0)	5 (12.3)	50 (5.7)	5 (2.7)	30 (7.3)
3 (11.7)	20 (6.7)	3 (38.0)	20 (6.0)	3 (3.0)	10 (33.0)	100 (8.3)	10 (7.3)	60 (7.7)
30 (29.0)	50 (9.7)	30 (65.0)	50 (12.7)	30 (4.0)	20 (35.7)	200 (9.3)	20 (31.0)	135 (8.7)
45 (38.0)	100 (11.6)	45 (84.7)	75 (27.5)	45 (5.3)	50 (37.3)	250 (16.2)	50 (64.1)	240 (11.7)
60 (59.3)	150 (35.3)	60 (95.0)	100 (43.6)	60 (11.0)	100 (39.0)	300 (24.3)	75 (77.7)	480 (38.6)
90 (83.3)	200 (61.7)	90 (99.0)	150 (51.7)	90 (38.7)	150 (52.3)	500 (58.7)	100 (95.7)	700 (70.7)
135 (98.7)	250 (83.0)	135 (99.3)	200 (80.0)	135 (98.0)	200 (81.0)	700 (83.8)	150 (99.3)	1000 (75.0)
240 (100)	300 (100)	240 (100)	250 (100)	240 (100)	250 (100)	1000 (97.7)	200 (100)	1200 (100)
Pb								
0 (0)	0 (0)	0 (0)	0 (0)	0 (0)	0 (0)	0 (0)	0 (3.0)	0 (0)
1 (1.3)	1 (4.0)	1 (10.3)	1 (19.7)	1 (6.0)	25 (2.3)	5 (4.3)	1 (4.5)	10 (6.0)
5 (5.7)	5 (5.7)	5 (11.3)	5 (29.0)	5 (7.3)	50 (6.0)	10 (5.3)	5 (6.5)	50 (8.0)
10 (13.1)	10 (11.3)	10 (14.0)	10 (33.0)	10 (6.7)	100 (10.3)	30 (6.7)	10 (10.0)	100 (7.3)
20 (22.9)	20 (11.7)	20 (27.0)	30 (36.0)	20 (7.8)	150 (14.2)	50 (11.0)	20 (13.4)	200 (15.7)
30 (32.3)	30 (18.7)	30 (38.7)	50 (54.7)	30 (10.0)	200 (34.3)	75 (14.7)	30 (33.7)	400 (19.3)
50 (39.4)	50 (32.6)	50 (55.3)	75 (73.7)	50 (15.3)	300 (74.7)	100 (27.0)	50 (74.2)	600 (20.1)
75 (73.7)	75 (56.9)	75 (66.0)	100 (87.0)	75 (79.3)	400 (95.6)	200 (55.3)	75 (93.1)	800 (70.7)
100 (98.0)	100 (95.0)	100 (99.3)	150 (97.7)	100 (98.3)	600 (100)	400 (99.0)	100 (95.9)	1000 (97.7)
Hg								
0 (1.7)	0 (0.0)	0 (0.7)	0 (0.3)	0 (0)	0 (1.3)	–	0 (0)	0 (0.7)
1 (20.5)	1 (0.3)	1 (11.4)	1 (4.4)	10 (0.3)	50 (32.1)		1 (4.0)	20 (0.7)
10 (27.6)	10 (7.7)	10 (29.9)	10 (16.1)	20 (8.0)	100 (34.1)		10 (12.3)	40 (1.7)
20 (28.7)	20 (13.8)	20 (34.6)	20 (18.7)	50 (10.7)	150 (37.5)		20 (47.0)	70 (2.4)
30 (32.1)	30 (20.1)	30 (52.3)	30 (35.4)	100 (38.0)	200 (53.7)		30 (85.7)	100 (5.2)
40 (38.9)	40 (23.2)	40 (63.8)	40 (52.5)	150 (43.0)	250 (57.1)		40 (94.2)	200 (11.8)
50 (53.9)	50 (62.4)	50 (87.9)	50 (78.3)	300 (71.0)	300 (77.0)		50 (94.9)	500 (38.8)
70 (74.4)	70 (87.6)	70 (97.0)	70 (95.7)	500 (98.7)	400 (97.9)		70 (99.0)	1000 (87.2)
100 (97.3)	100 (98.7)	100 (100.0)	100 (100)	1000 (100)	600 (100)		100 (100)	1200 (98.3)

n = 3, – not studied

The data show that the germination process in the plants listed above is most significantly inhibited at metal concentrations ≥ 10 mg/L. The most sensitive plants reacted by reducing their germination capacity by 20% already at metal concentrations of about 1 mg/L, e.g. rye (Table 2).

The species showing the highest resistance to the studied metals (except Cu) was vetch (Tables 2, 3). A 20% reduction in germination capacity was observed in this species at very high metal concentrations, from 200 to 350 mg/L (Ni) to as much as 500–750 mg/L (Zn). The effect of heavy metals on germination is due to the fact that they impede water penetration into seeds, which results in a low swelling rate and, in consequence, a lower rate and degree of germination (compared to the control) (Rasafi et al. 2016). A delayed germination of seeds subjected to higher concentrations of metals as compared to the control was also observed in the current study. Depending on the seed species and metal ions, the duration of the test was extended up to 7 days (e.g. pea).

**Fig. 1:**
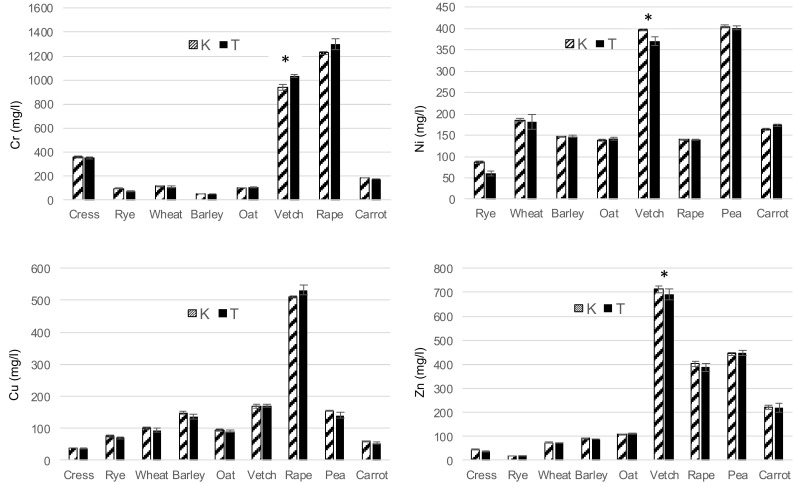
Acute lethal toxicity (LC50) of Cr, Ni, Cu, Zn to plants according Kraber’s (K) and Trevan’s (T) methods. All values are expressed as mean ± SE of mean; asterisk indicates statistically significant difference between the Kraber and Trevan’s methods (*p* < 0.05, Duncan’s test comparison of means)

**Fig. 2:**
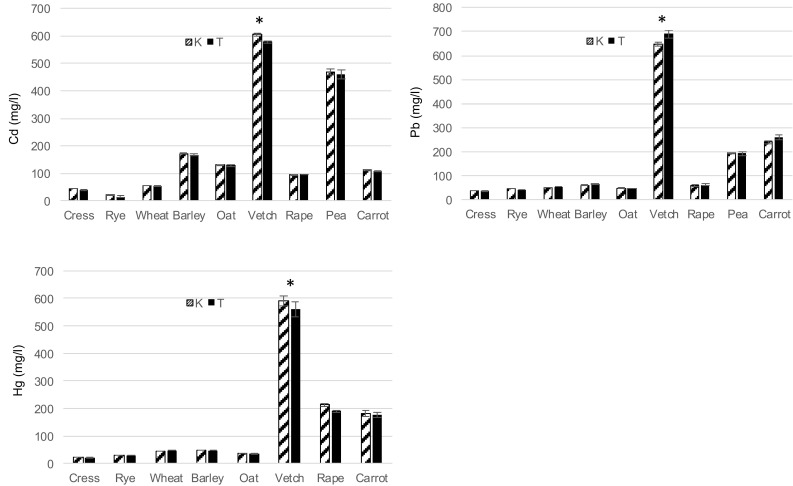
Acute lethal toxicity (LC50) of Cd, Pb, Hg to plants according Kraber’s (K) and Trevan’s (T) methods. All values are expressed as mean ± SE of mean; asterisk represents statistically significant difference between the Kraber and Trevan’s methods (*p* < 0.05, Duncan’s test comparison of means)

Based on the results presented in Tables 2 and 3, LC50 values were calculated using Kraber’s (K) and Trevan’s (T) methods (Figs. 1, 2). In most cases, mean LC50 values did not differ significantly between both methods. Significant differences (*p* ≤ 0.05) were only shown by Duncan’s test in the case of species with the lowest sensitivity, i.e. vetch (Cr, Ni, Zn, Cd, Pb, Hg) and rape (Cr, Hg). For metals such as Zn, Cd, Pb and Hg, statistically the highest LC50 values were obtained for vetch, which proves that of all the studied species, it has the highest resistance to these metal ions. Furthermore, vetch and the seeds of rape, cress and rye exhibited the lowest response to chromium ions, vetch and pea seeds – to nickel, and barley and pea – to cadmium ions. A study conducted by Fargašová (1994) demonstrated the following order of toxicity for metal ions on the germination of *Sinapis alba* seeds: As > Cr = Hg > Cd > Pb based on LC50 values. In this study, a similar toxicity of Hg, Pb and Cd was observed in wheat and vetch. The degree of metal toxicity determined on the basis of LC50 values, in relation to all the investigated plant seeds, is presented in the scheme below:

Cr: rape < vetch < cress < carrot < wheat < oat < rye < barley; Cu: rape < vetch < pea < barley < wheat < oat < rye < carrot < cress; Ni: pea < vetch < wheat < carrot < barley < oat < rape < rye; Zn: vetch < pea < rape < carrot < oat < barley < wheat < cress < rye; Cd: vetch < pea < barley < oat < carrot < rape < wheat < cress < rye; Pb: vetch < carrot < pea < barley < rape < wheat < oat < rye < cress; Hg: vetch < rape < carrot < wheat < barley < oat < rye < cress.

In the present study, a significant decrease in seed germination was observed in the range from 50 mg/L (cress, rye, oat) to even 800 mg/L (vetch). The study shows that vetch seeds are characterized by the best tolerance to almost all of studied metal ions (except for Cu). A comparable seed resistance to high concentrations of Ni and Cd was also observed in peas, and in the case of Cr – in rape. These species could be considered potentially suitable for the reclamation of areas with high concentrations of these metals.

## References

[CR1] El-Ghamery AA, El-Kholy MA, El-Yousser MAA (2003). Evaluation of cytological effects of Zn^2+^ in relation to germination and root growth of *Nigella sativa* L. and *Triticum aestivum* L. Mutat Res.

[CR2] Fargašová A (1994). Effect of Pb, Cd, Hg, As, and Cr on germination and root growth of *Sinapis alba* seeds. Bull Environ Contam Toxicol.

[CR3] Kranner I, Colville L (2011). Metals and seeds: Biochemical and molecular implications and their significance for seed germination. Environ Exp Bot.

[CR4] Li W, Khan MA, Yamaguchi S, Kamiya Y (2005). Effects of heavy metals on seed germination and early seedling growth of *Arabidopsis thaliana*. Plant Growth Regul.

[CR5] Peralta JR, Gardea-Torresdey JL, Tiemann KJ, Gomez E, Arteaga S, Rascon E, Parsons JG (2001). Uptake and effects of five heavy metals on seed germination and plant growth in alfalfa (*Medicago sativa* L.). Bull Environ Contam Toxicol.

[CR6] Rasafi TE, Nouri M, Bouda S, Haddioui A (2016). The effect of Cd, Zn and Fe on seed germination and early seedling growth of wheat and bean. Ekológia (Bratislava).

[CR7] Regulation of the Minister of the Environment (2016) pos. 1395, on the way of assessing the pollution of the earth’s surface

[CR8] Saganuwan SA (2016). Toxicity studies of drugs and chemicals in animals: an overview. Bulg J Vet Med.

[CR9] Sethy SK, Ghosh S (2013). Effect of heavy metals on germination of seeds. J Nat Sci Biol Med.

[CR10] Smreczak B, Maliszewska-Kordybach B (2003). Seeds germination and root growth of selected plants in PAH contaminated soil. Fresen Environ Bull.

[CR11] Statistical Yearbook of Agriculture (2016) https://stat.gov.pl/files/gfx/portalinformacyjny/en/def

[CR12] Tangahu BV, Abdullah SRS, Basri H, Idris M, Anuar N, Mukhlisin M (2011). A review on heavy metals (As, Pb, and Hg) uptake by plants through phytoremediation. Hindawi Publishing Corporation. Int J Chem Eng.

[CR13] Tao L, Guo M, Ren J (2015). Effects of cadmium on seed germination, coleoptile growth, and root elongation of six pulses. Pol J Environ Stud.

[CR15] Zhou H, Zhou X, Zeng M, Liao BH, Liu L, Yang WT, Qiu QY, Wang YJ (2014). Effects of combined amendments on heavy metal accumulation in rice (*Oryza sativa* L.) planted on contaminated paddy soil. Ecotox Environ Safe.

